# Whole-Genome Sequencing Identifies the Egl Nine Homologue 3 (egln3/phd3) and Protein Phosphatase 1 Regulatory Inhibitor Subunit 2 (PPP1R2P1) Associated with High-Altitude Polycythemia in Tibetans at High Altitude

**DOI:** 10.1155/2019/5946461

**Published:** 2019-11-07

**Authors:** Luobu Gesang, Lamu Gusang, Ciren Dawa, Gawa Gesang, Kang Li

**Affiliations:** ^1^High Altitude Medical Research Institute of Tibet, Lhasa 850000, China; ^2^Department of Cardiology, People's Hospital of Tibet Autonomous Region, Lhasa 850000, China

## Abstract

**Background:**

The hypoxic conditions at high altitudes are great threats to survival, causing pressure for adaptation. More and more high-altitude denizens are not adapted with the condition known as high-altitude polycythemia (HAPC) that featured excessive erythrocytosis. As a high-altitude sickness, the etiology of HAPC is still unclear.

**Methods:**

In this study, we reported the whole-genome sequencing-based study of 10 native Tibetans with HAPC and 10 control subjects followed by genotyping of selected 21 variants from discovered single nucleotide variants (SNVs) in an independent cohort (232 cases and 266 controls).

**Results:**

We discovered the egl nine homologue 3 (egln3/phd3) (14q13.1, rs1346902, *P* = 1.91 × 10^−5^) and PPP1R2P1 (Protein Phosphatase 1 Regulatory Inhibitor Subunit 2) gene (6p21.32, rs521539, *P* = 0.012). Our results indicated an unbiased framework to identify etiological mechanisms of HAPC and showed that egln3/phd3 and PPP1R2P1 may be associated with the susceptibility to HAPC. Egln3/phd3b is associated with hypoxia-inducible factor subunit *α* (HIF*α*). Protein Phosphatase 1 Regulatory Inhibitor is associated with reactive oxygen species (ROS) and oxidative stress.

**Conclusions:**

Our genome sequencing conducted in Tibetan HAPC patients identified egln3/phd3 and PPP1R2P1 associated with HAPC.

## 1. Introduction

In excess of 140 million humans have primarily lived in the high-altitude regions of various locations around the world, including the Qinghai-Tibet plateau in Asia, the Andes Mountains of South America, and the Ethiopian plateau in East Africa [1]. All dwellers in plateaus like native Tibetans of the Qinghai-Qinghai-Tibet plateau are regarded as one of the best dwellers adapted to high altitudes. They possess heritable adaptations to the hypoxic condition [2], as they display lower hemoglobin and hematocrit [3], higher oxygen saturation of blood [4], and better work efficiency [5], which are conducive to adapting the high-altitude and hypoxic situations. However, some Tibetans living at high altitude with genetic adaptations remain maladapted and possess symptoms of high-altitude polycythemia (HAPC).

HAPC is diagnosed with excessive erythrocytosis encountered by around 10% of the population residing at the Qinghai-Tibet plateau [6, 7]. HAPC shows several impacts on blood viscosity with various harmful consequences such as dysfunction of microcirculation, vascular thrombosis, extensive organ damage, sleep disturbances, and even death [7, 8]. In high-altitude dwellers, hypobaric hypoxia is thought to be one of the primary initiators of HAPC. However, the mechanisms underlying the pathogenesis and genetic basis of HAPC have not been well understood.

In recent years, the genetic basis underlying adaptation to high altitude in Tibetans has been elucidated by using exome and single nucleotide polymorphism (SNP) array data [9–11]. Furthermore, gene candidate approaches in Tibetan have identified that genomic variants in *EPAS1*, *CYP17A1*, *CYP2E1*, and mitochondrial DNA 10609T are associated with HAPC [12–15]. However, comprehensive genome-wide scanning to detect the variations associated with HAPC has not been performed. It has been known that the variants that occurred in the genome-wide association study (GWAS) may account for some diseases with only relatively modest proportions and numerous deleterious variants with low frequency in the population.

With the technical development of high-throughput sequencing, it provides an opportunity to resequence multiple genetic regions and recognize novel risk alleles in diseases including high-altitude sickness [1, 16–18]. To dissect the genetic mechanisms underlying HAPC, we compared genetic variations between native Tibetans with HAPC and adapted subjects without HAPC by whole-genome sequencing (WGS) of 20 individuals, and selected variants were further genotyped in an additional larger case-control cohort. Our investigation revealed the egl nine homologue 3 (egln3/phd3) (14q13.1) and PPP1R2P1 (Protein Phosphatase 1 Regulatory Inhibitor Subunit 2) gene (6p21.32) associated with HAPC.

## 2. Methods

### 2.1. Patients and Healthy Control Subjects

As the same ethnicity, HAPC was collected in healthy control subjects and matched with native high-altitude Tibetans. In this study, 10 patients with HAPC and 10 control subjects without HAPC that were performed in Tibet area (Table 1) were recruited. Based on the International Consensus Statement [7], (1) HAPC was diagnosed with an Hb concentration of at least 19 g/dL and 21 g/dL for women and men, respectively, and (2) HAPC patients were local Tibetans normally living at 3600 to 4500 m. Blood samples for WGS were collected, and each subject was informed and gave written consent.

### 2.2. Whole-Genome Sequencing

Blood DNA was isolated from each subject, and the desired length fragments were gel purified. According to the manufacturer's instruction, DNA cluster preparation and adaptor ligation were performed with the library preparation kit (Illumina). WGS was performed on Illumina HiseqX ten Platform with 150 base pairs (bps) paired-end reads.

### 2.3. Read Alignment and Variant Calling

All reads were mapped to human reference genome by BWA [19] with default parameter. For downstream filtering, picard tools (http://broadinstitute.github. io/picard/) were used to mark PCR duplicates. We used Genome Analysis Toolkit (GATK) to adjust the alignments via GATK indel realignment and score recalibration modules [20]. We finally called and filtered SNVs using the GATK Unified Genotyper under default parameters. With the Ensembl annotated database, the resulting SNVs were annotated by ANNOVAR [21] according to the locations and expected effect on encoded gene products. According to dnSNP, SNVs were identified into “newly identified” and “known” variants.

### 2.4. SNP Discovery Stage

The allele frequency distribution of 10 cases and 10 controls and variants with significant difference (*P* < 0.01) were analyzed by the Fisher exact test in allele frequency. To select the SNVs for association mapping, the pathway enrichment analysis using WebGestalt (http://bioinfo.vanderbilt.edu/webgestalt/) was performed by the distribution of genes with different allele frequencies. We used the hypergeometric test and Benjamini-Hochberg FDR to determine the significance of enrichment and adjust the multiple testing, respectively. This analysis revealed pathways including the HIF-1 signaling pathway and hematopoietic cell lineage [22] were associated with high-attitude adaption. We selected 21 SNVs in the two pathways from SNVs with different allele frequencies for genotyping in the stage of the association study.

### 2.5. Genotyping and Statistical Analysis

Genotyping of the selected 21 variants was performed with Sequenom MassARRAY or Snapshot system in an additional 232 HAPC cases and 266 controls. The primer of genotyping was designed by use of Primer 5.0. The frequency of alleles was calculated by genotype frequency in HAPC patients and control subjects, and the intergroup differences were counted using the Fisher exact test. *P* value for multiple testing was adjusted by Benjamini-Hochberg FDR. QQ plot was produced using the man statistics package of R (http://cran.r-project.org/web/packages/qqman/). We use the chi-square test to assess deviations from Hardy-Weinberg equilibrium.

## 3. Results

### 3.1. Whole-Genome Sequencing and Single Nucleotide Variants (SNVs)

After extracting DNA from the peripheral blood, the whole-genome sequencing of 10 native Tibetans with HAPC and 10 adapted subjects without HAPC was performed by using next-generation sequencing technology (Supplementary [Supplementary-material supplementary-material-1]). As a result, there were more than 360 million paired-end reads for each subject (Table 2). More than 99.7% of the reference genome was covered, and we sequenced the genome with a mean depth of 39x per individual. After applying stringent quality controls, we obtained 69,591,555 SNVs (single nucleotide variants), of which 191,734 (0.28%) were nonsilent mutations (nonsynonymous and splice site mutations). Comparing with 1000 Genome Project [23] gave 94% of SNVs known in 1000 Genomes Phase 1 ASN.

To discover the susceptibility loci associated with HAPC, two-stage studies including SNP discovery study and replication study were performed. To select the candidate SNVs for association mapping, we applied the Fisher exact test to WGS data and identified 33,662 SNVs (*P* < 0.01) in allele frequency between native Tibetans with HAPC and adapted subjects without HAPC, of which 72 were nonsilent SNVs (Supplementary [Supplementary-material supplementary-material-1]). Pathway enrichment analysis was performed on the genes with significant difference in allele frequency and revealed pathways including the HIF-1 signaling pathway [24] and hematopoietic cell lineage [22] that were associated with high-attitude adaption (Supplementary [Supplementary-material supplementary-material-1]). To explore the association between HAPC and the two pathways, we subjected 21 SNVs that are in the two pathways and also showed allele frequency difference in the above Fisher exact test for genotyping in the stage of the association study (Supplementary [Supplementary-material supplementary-material-1]).

### 3.2. Association Study

**Figure 1:**
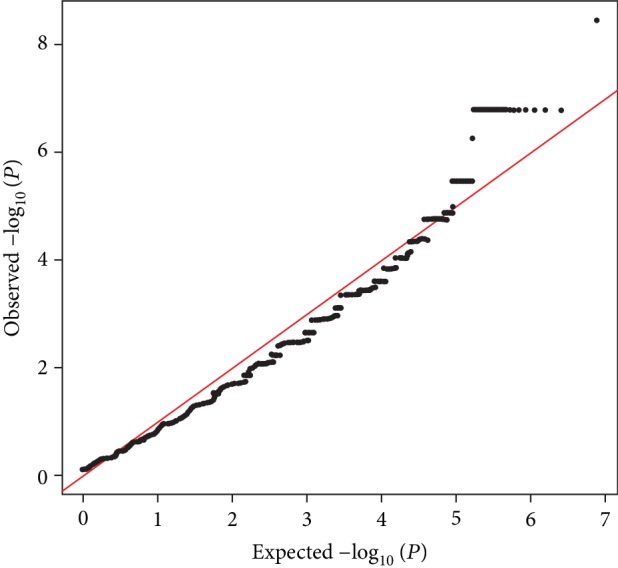
QQ plot indicates the differences between observed and expected -log_10_ (*P* value).

Associations between the 21 selected variants and HAPC were evaluated by genotyping in an additional 232 Tibetans with HAPC and 266 control subjects (Supplementary [Supplementary-material supplementary-material-1]). Most of the observed associations showed close to the expected distribution based on the quantile-quantile (QQ) plot, which is consistent with the null hypothesis of no association (Figure 1). Two loci exceeded significance in association with HAPC (Table 3): egln3/phd3 at rs1346902 in *EGLN3* on 14q13.1 (*P* = 1.91 × 10^−5^, odds ratio (OR) = 0.57, 95% confidence intervals (CI): 0.44-0.74) and PPP1R2P1 at rs521539 in the region between *HLA-DRB1* and *HLA-DQA1* on 6p21.32 (*P* = 0.012, OR = 1.41, 95% CI: 1.08-1.84). The allele frequencies of other variants between these two groups were not significantly different. Furthermore, the overlap of these two associated SNPs was evaluated with the Encyclopedia of DNA Elements—annotated genomic elements (Supplementary [Supplementary-material supplementary-material-1]). There were histone modifications at promoter or enhancer; DNase hypersensitivity sites, binding proteins, and motifs changed in both SNVs.

### 3.3. The Analysis of Clinical and Genetic Correlation

**Figure 2:**
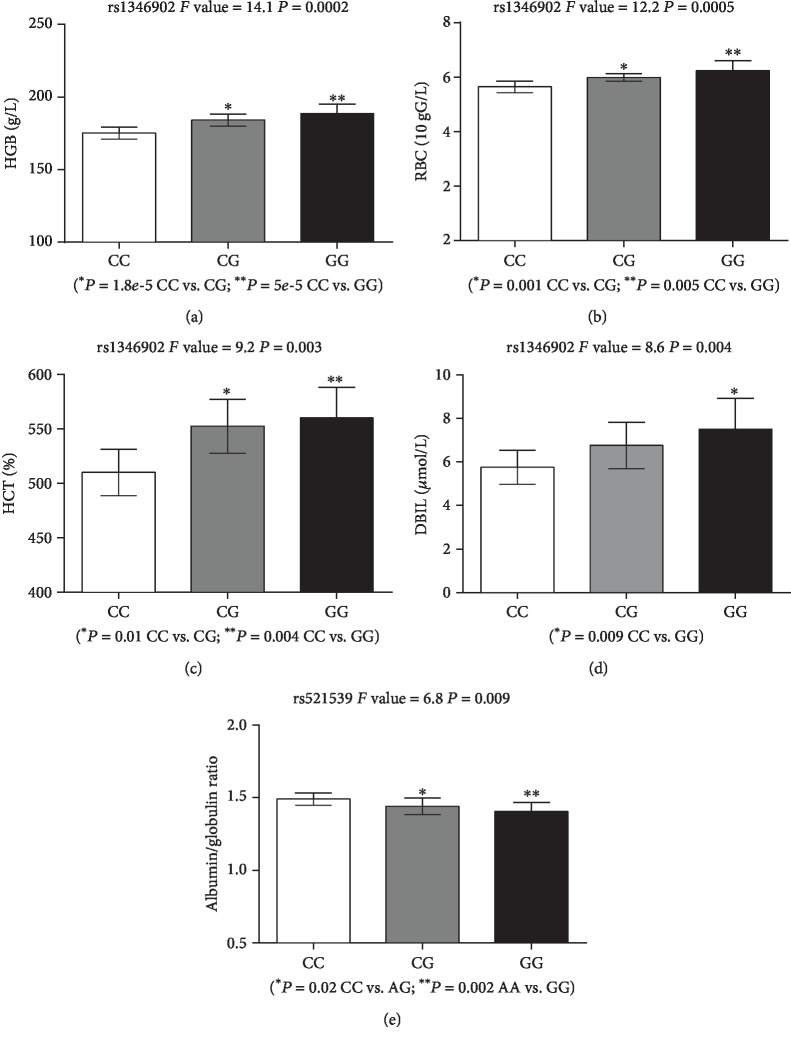
Clinical and genetic association analysis for the egl nine homologue 3 (egln3/phd3) (14q13.1, rs1346902, *P* = 1.91 × 10^−5^) and PPP1R2P1 (Protein Phosphatase 1 Regulatory Inhibitor Subunit 2) gene (6p21.32, rs521539, *P* = 0.012). Significant correlation was found between AX9 and NKX2-1 and hemoglobin (HGB or Hb) (a), red blood cell count (RBC) (b), hematocrit (HCT) (c), and direct bilirubin (DBIL) (d). PPP1R2P1 was also significantly correlated with the albumin to globulin ratio (e). The association analysis was examined by analysis of variance method, and differences between genotypes were evaluated with a *t*-test.

We next measured whether tegln3/phd3 and PPP1R2P1 were concerned with clinical characters between the HAPC group and the control group using analysis of variance method (Figure 1). In all Tibetan subjects, we found the obvious correlation (*P* < 0.01) between tegln3/phd3 and hemoglobin (HGB or Hb; *F* value = 14.1, *P* = 0.0002), red blood cell count (RBC; *F* value = 12.2, *P* = 0.0005), hematocrit (HCT; *F* value = 9.2, *P* = 0.003), and direct bilirubin (DBIL; *F* value = 8.6, *P* = 0.004). Tibetan subjects with CC genotype showed lower Hb than those with either the CG (*P* = 1.8 × 10^−5^) or the GG (*P* = 5 × 10^−5^) genotype (Figure 2(a)). Similarly, the lower HGB, HCT, and DBIL were also observed in individuals with CC genotype compared with CG or GG individuals (Figures 2(b)–2(d)). Additionally, we also found a significant association between PPP1R2P1 (A/G) and the albumin to globulin ratio (Figure 2(e); *F* value = 6.8, *P* = 0.009).

## 4. Discussion

In the present study, we used whole-genome sequencing of native Tibetans with HAPC and adapted subjects without HAPC to identify alleles' associated risk in previously unidentified HAPC susceptibility genes. We selected 21 variants from our WGS data for further evaluation in an additional larger cohort of Tibetans with HAPC and controls. The differences in the SNPs of tegln3/phd3 (C/G) and PPP1R2P1 (A/G) between the two groups are found for the first time in this present paper.

SNP rs1346902 is located in the intron region (transcript ID: ENST00000487915 and ENST00000551935) of *EGLN3* (also known as PHD3) that belongs to the Egl-9 (EGLN) family. *EGLN3*, along its paralogous genes *EGLN1* and *EGLN2*, can catalyze the hydroxylation of hypoxia-inducible factor subunit *α* (HIF*α*), leading to increased ubiquitination and proteasomal degradation of HIF*α* and decreased HIF*α* activity [25]. Upregulated *EGLN3* is related to apoptosis in sympathetic neurons [26], p53-induced growth arrest in RAS-transformed embryo fibroblasts [27], and differentiation of C2C12 myoblasts [28]. *EGLN3* also plays important roles in human endothelial cells [29] and cancers, including pancreatic cancer [30] and glioblastoma [31]. Previous study also identifies that *EGLN3* can negatively regulate the NF-*κ*B pathway, which is a crucial regulator of cell differentiation, inflammation, stress responses, and immunity [32]. Although association analysis with microsatellite markers in an Andean high-altitude population shows a possible association between *EGLN3* (marker D14S1049) and severe polycythemia [33], no susceptible association between SNPs in *EGLN3* and HAPC is reported. Here, we found a susceptibility locus in *EGLN3* for HAPC. We also found significantly lower Hb and RBC in the subjects with rs1346902 C/C allele (Figures 2(a) and 2(b)), supporting and extending the potential role of *EGLN3* in HAPC. Therefore, the significant difference of genotypes between two groups for SNP rs1346902 (C/C) was the beneficial allele of HAPC and it might contribute to high-altitude adaption in Tibetans with lower Hb and RBC.

A large number of genomic surveys about nucleotide polymorphism among humans in high-altitude environments have offered evidence for genes in the HIF oxygen signaling pathway, among which *EPAS1* and *EGLN1* are notable [9, 10, 34–36]. *EPAS1*, *EGLN1*, *VEGFA*, *CYP17A1*, and *CYP2E1* in the HIF pathway are also shown to be associated with high-altitude sickness, including HAPC [12–14, 37–39]. The finding of susceptibility SNP in *EGLN3* for HAPC in our study further supported the key role of the HIF oxygen signaling pathway in high-altitude adaption and relevant diseases.

Human leukocyte antigen (HLA) loci have been associated with more than 100 diseases [40]. *HLA-DRB1* and *HLA-DQA1* play an important role in the immune system, which belong to the classical HLA class II molecules. They can take part in recognizing and presenting antigens to T cells and are mainly expressed in B cells, dendritic cells, and macrophages. In addition, several studies have found a connection between HLA class II molecules and immune-mediated disorders, such as susceptibility loci in *HLA-DRB1* for sarcoidosis [41] and pemphigus [42] and susceptibility loci in *HLA-DQA1* for achalasia [43] and celiac diseases [44].

In our study, we also found that there were significant differences (*P* = 0.012) in the allele frequency of PPP1R2P1 near *HLA-DRB1/DQA1* between the HAPC group and the control group, with high-frequency A allele among HAPC (57%) than controls (47%). Our finding was consistent with positive selection of HLA loci (*HLA-DRA*, *HLA-DQB1/HLA-DQA2*) in Ethiopian population [45] and significantly associated with the high-altitude pulmonary edema of *HLA-DR6* and *HLA-DQ4* [46]. Our results also revealed a significant correlation between PPP1R2P1 (A/G) and albumin/globulin ratio (Figure 2(e)), which is an indicator of liver and kidney disorders. The implication of HLA suggests a role of inflammation perhaps associated with the kidneys or livers. Previous studies show that chronic hypoxia induced hypertension and erythrocytosis leads to renal injury and inflammation in rats [47]. The similar effect between HLA and HAPC may exist in humans. Protein Phosphatase 1 Regulatory Inhibitor has been reported to be associated with reactive oxygen species (ROS) and oxidative stress [48].

The primary limitation of our research was the small case-control cohort in the discovery phase, which might result in low power of detection of candidate susceptibility loci. Moreover, the functional role of newly identified susceptibility loci was needed to be verified by some experiments. We will pay more attention to the expression pattern of these genes and related regulatory pathways in HAPC patients.

## 5. Conclusions

In summary, our genome sequencing conducted in Tibetan HAPC patients identified two new susceptibility loci at 14q13.1 and 6p21.32 and offered to our knowledge of the genetic architecture of HAPC. Further study about these two loci was likely to improve our knowledge on the etiology of HAPC.

## Figures and Tables

**Table 1:** Clinical characterization between two groups.

Group	Sample	Gender	Age	Living place	Living altitude	Systolic blood pressure	Diastolic blood pressure	Hb
HAPC	D1WGC025333	F	56	Lhasa	3650 m	120	80	235
	D2WGC025334	M	46	Xigaze	3900 m	120	80	244
	D3WGC025335	M	62	Lhasa	3650 m	120	80	220
	D4WGC025336	M	59	Naqu	4500 m	120	80	274
	D5WGC025337	M	37	Xigaze	3900 m	130	110	230
	D6WGC025338	M	30	Xigaze	3900 m	120	70	219
	D8WGC025340	M	34	Shannan	3500 m	100	60	215
	D10WGC025341	M	48	Shannan	3500 m	124	80	249
	D13WGC025344	M	36	Shannan	3500 m	116	70	237
	D15WGC025346	M	46	Linzhi	3000 m	100	60	240

Control	Z1WGC025347	F	60	Xigaze	3900 m	110	70	140
	Z2WGC025348	M	64	Xigaze	3900 m	120	80	146
	Z3WGC025349	F	50	Lhasa	3650 m	120	80	163
	Z4WGC025350	M	52	Xigaze	3900 m	120	80	162
	Z5WGC025351	M	63	Shannan	3500 m	120	80	162
	Z6WGC025352	F	42	Xigaze	3900 m	116	76	146
	Z7WGC025353	M	41	Xigaze	3900 m	130	90	163
	Z8WGC025354	M	48	Shannan	3500 m	110	82	162
	Z13WGC025359	M	44	Shannan	3500 m	102	56	174
	Z14WGC025360	F	65	Xigaze	3900 m	120	80	170

Note: HAPC: high-altitude polycythemia; M: male; F: female.

**Table 2:** Summary of whole-genome sequencing and variant call statistics per individual.

Category	Sample	Paired-end reads (million)	Mapped rate	Mean depth	Coverage of genome	Final SNVs	SNVs (%)	Nonsilent SNVs
HAPC	D1WGC025333	421.0	99.6%	42.5	99.7%	2,672,708	94.2	6,600
	D2WGC025334	417.6	99.5%	41.8	99.8%	3,506,627	94.1	9,610
	D3WGC025335	404.4	99.7%	41.1	99.8%	3,536,613	93.5	9,926
	D4WGC025336	408.8	99.5%	41.3	99.7%	3,532,401	93.7	9,918
	D5WGC025337	410.3	99.6%	41.6	99.8%	3,511,589	94.2	9,605
	D6WGC025338	402.3	99.5%	40.7	99.7%	3,507,517	94.1	9,595
	D8WGC025340	366.2	40.7%	14.4	99.5%	3,362,350	93.2	9,593
	D10WGC025341	412.0	97.4%	40.7	99.8%	3,529,927	93.6	9,760
	D13WGC025344	392.8	99.5%	39.3	99.8%	3,538,731	93.5	9,860
	D15WGC025346	401.3	99.4%	40.3	99.7%	3,510,739	93.8	9,687

Control	Z1WGC025347	429.5	99.5%	43.5	99.1%	3,546,349	93.5	9,750
	Z2WGC025348	365.3	99.8%	37	99.7%	3,529,029	93.4	9,835
	Z3WGC025349	305.2	99.6%	30.9	99.0%	3,561,607	93.3	9,940
	Z4WGC025350	429.0	99.6%	43.4	99.7%	3,511,388	93.7	9,629
	Z5WGC025351	422.8	99.6%	42.6	99.7%	3,513,151	93.8	9,467
	Z6WGC025352	363.9	99.8%	37	99.1%	3,544,574	93.6	9,735
	Z7WGC025353	412.8	99.5%	41.8	99.8%	3,535,839	93.6	9,857
	Z8WGC025354	420.8	99.4%	42.7	99.7%	3,544,492	93.6	9,705
	Z13WGC025359	413.5	99.5%	41.9	99.8%	3,534,525	93.5	9,841
	Z14WGC025360	412.1	99.5%	41.9	99.1%	3,561,399	93.4	9,821

**Table 3:** Association between HAPC and 2 variants.

MAF							
Chr.	Near gene	Variants	Allele	Case	Control	*P* value	OR (95% CI)
14q13.1	EGLN3	rs1346902	C/G	0.342	0.477	0.0000191	0.57 (0.44-0.74)
6p21.32	HLA-DRB1-HLA-DQA1	rs521539	A/G	0.561	0.475	0.012	1.41 (1.08–1.84)^∗^

Chr.: chromosome; MAF: minor allele frequency; CI: confidence interval. Alleles are shown as minor allele/major allele. ^∗^Odds ratio and CI for A allele vs. G allele. The egl nine homologue 3 (egln3/phd3) (14q13.1, rs1346902, *P* = 1.91 × 10^−5^) and PPP1R2P1 (Protein Phosphatase 1 Regulatory Inhibitor Subunit 2) gene (6p21.32, rs521539, *P* = 0.012).

## Data Availability

Our datasets are presented within the manuscript and additional supporting files.

## Abbreviations

HAPC: High-altitude polycythemia
SNP: Single nucleotide polymorphism
GWAS: Genome-wide association study
WGS: Whole-genome sequencing
SNVs: Single nucleotide variants
HIF*α*: Hypoxia-inducible factor subunit *α*
HLA: Human leukocyte antigen.

## Supplementary Materials

Supplementary Materials Supplementary Table S1: sequence data for HAPC and control subjects. Supplementary Table S2: list of 72 nonsilent SNPs with *P* < 0.01 in allele frequency between cases and healthy controls in the discovery phase. Supplementary Table S3: pathway enrichment analysis on the genes with significant difference in allele frequency between 10 cases and 10 controls. Supplementary Table S4: the selected 21 SNPs for genotyping. Supplementary Table S5: characteristics of case and control subjects in the genotyping phase. Supplementary Table S6: functional annotation of SNPs correlated with rs1346902 and rs521539 (*r*^2^ > 0.8) using data from ENCODE.
